# Drying out fish ponds, for an entire growth season, as an agroecological practice: maintaining primary producers for fish production and biodiversity conservation

**DOI:** 10.12688/openreseurope.16363.2

**Published:** 2024-10-15

**Authors:** Léo GIRARD, Alexander WEZEL, Joël ROBIN

**Affiliations:** 1 Agroecology and Environment Research Unit, ISARA, Lyon, 69364, France

**Keywords:** macrophytes, fishpond, shallow lake, sediment, agroecology, disturbance

## Abstract

Agroecology largely focusses on terrestrial agroecosystems, but it can also be applied to fish farming. Indeed, ponds are typically used for fish production in Europe, but are also important reservoirs of biodiversity. Numerous studies demonstrate that both fish production and biodiversity are strongly determined by human management. One key practice in extensive fish farming, although more rare in Europe, is to dry out ponds. They are left dry for a complete year after several years of fish production. However, the extent to which this practice affects the functioning of the ecosystem, its biodiversity and fish production remain unclear.

We investigated data from 85 fish ponds in the Dombes region, France, sampled between 2007 and 2014. We related variation in key abiotic characteristics to the time since last dry out. The dataset included organic matter content in pond sediments and concentrations of inorganic nitrogen and phosphorus in the water column, and biotic components such as macrophytes cover and richness, phytoplankton concentration and richness, macroinvertebrates density, and fish yield.

Our results show that drying out facilitates the mineralization of organic matter in sediments and results in higher concentrations of inorganic nitrogen in the water column. Macrophytes cover is highest during the first year after drying out, and gradually declines after at the expense of increasing phytoplankton concentration. The diversity of both is highest in the first year after drying out and declines rapidly, especially for macrophytes. Fish yield is at its maximum in the second year.

Drying out fish ponds appears to be an important agroecological practice in extensive fish farming with an application every three to four years. By nutrient recycling, this practice has a positive impact on the balance between primary producers and indirectly on the whole food web during two years. It optimizes fish production and allows biodiversity conservation.

## Introduction

Agricultural landscapes in Europe are considered as important habitats for biodiversity conservation in anthropogenic areas ( Eriksson, 2021; Queiroz *et al*., 2014). They are therefore increasingly targeted by nature protection policies that aim to maintain or increase biodiversity. For this purpose, multiple areas in Europe have been classified as Natura 2000 regions or as High Nature Value (HNV) areas ( Andersen *et al*., 2004; Evans, 2012; Mueller & Maes, 2015). Agricultural landscapes are typically subjected to human interference to promote agricultural production and their ecological status strongly relies on specific practices ( Schmitzberger *et al*., 2005).

Several agricultural landscapes Europe are characterized by the occurrence of a high density of man-made ponds that have historically been used for extensive fish farming. Examples of such pond landscapes, also called “pondscapes”, are Midden-Limburg in Belgium, Trebon in the Czech Republic, Lausitz, Aischgrund and Oberpfalz in Germany, Waldviertel in Austria, but also Dombes, Lorraine, Brenne and Forez in France ( Aubin *et al*., 2017). Many fish ponds have a rich history as they have often been created by monastic communities in the Middle Ages, in an effort to drain swampy areas and wetlands ( Avocat, 1975; Guichenon, 1650 cited in Sceau, 1980), to ensure agricultural production, to reduce diseases, and to promote the production of freshwater fishes (Guichenon, 1650 cited in Sceau, 1980). Today, extensive fish production is still an important agricultural practice in most of these regions. Pond management practices, including water management, pond fertilisation or liming, and fish stocking can vary strongly between regions and countries ( Horvàth *et al*., 2002). However, the overall activities largely target the production of cyprinid fish species, such as common carp ( *Cyprinus carpio*) for human consumption and restocking programs ( Horvàth *et al*., 2002).

All these ponds, including fish ponds, collectively contain more species and more rare species than other aquatic environments, such as lakes, rivers and canals ( Williams *et al*., 2004). In harbouring rare as well as endemic species, they form irreplaceable habitats ( Céréghino *et al*., 2007) and they play an important role in biodiversity conservation ( Biggs *et al*., 1994; Céréghino *et al*., 2007; Oertli *et al*., 2005; Williams *et al*., 2004). Although fish production and human management, fish ponds are no exception in this regard, in the same way as natural ponds ( Zamora-Marin *et al*., 2021). These areas represent a mosaic of diverse habitats and can be considered as hotspots of biodiversity ( Broyer & Curtet, 2012; Lemmens *et al*., 2013; Rosset *et al*., 2014; Vanacker *et al*., 2015; Wezel *et al*., 2013; Wezel *et al*., 2014). Fish ponds can be also home of rare or protected fauna and flora ( Vallod & Wezel, 2010; Wezel *et al*., 2013; Wezel *et al*., 2014). As mentioned previously, these agroecosystems are of human origin and thus artificial environments, associated with extensive fish farming practices ( Avocat, 1975; Guichenon, 1650 cited in Sceau, 1980). The biodiversity present in these agroecosystems is also the result of these applied historical practices ( Vallod & Wezel, 2010). Moreover, it appears that unmanaged ponds are not conducive to biodiversity ( Sayer *et al*., 2012)

Fish pond managers are therefore increasingly challenged to combine economically profitable fish farming activities with maintaining proper ecological ecosystem functioning and high levels of biodiversity in these agroecosystems. In these ‘pondscapes’, fish farmers play an important role in biodiversity conservation by maintaining ponds or by organizing water circulation in the pond networks. In this regard, they are dealing with agroecology applied to extensive fish farming. Agroecology is based on 13 principles ( HLPE, 2019; Wezel *et al*., 2020), some of which are applicable to aquaculture and fish pond agroecosystems management. According to Aubin *et al*. (2017), agroecological fish pond systems can be defined with the following five principles: It should be (1) productive, (2) resilient and robust, (3) efficient in the use of local resources, but also (4) environmentally friendly, and (5) have a natural and cultural value. Here we find together the notions of production, conservation of biodiversity, and ecological status of systems. Indeed, biological interactions and ecosystem synergies build a complex trophic web, which allow fish production. However, the literature on agroecological practices applied to pond fish farming remains limited.

A key management action in fish ponds is regular periodic drainage of ponds to harvest fish ( Horvath *et al*., 2002; Lierdeman, 2013). This is typically done from autumn to late winter (generally from October to March). The pond is refilled quickly after, in early spring, for a new production season. Lemmens *et al*. (2015) mentioned that this periodic pond drainage is important for biodiversity conservation. In addition to these short dry periods, in Dombes, ponds are empty every 4 or 5 years during a whole production season, approximately from March to September. It is an ancestral practice, called dry out in this paper, applied regularly and in some other fish pond systems ( Horvath *et al*., 2002; Lierdeman, 2013). Of all the extensive fish farming practices, drying out is the most common applied in the Dombes region. It is also a practice that brings together the most different ecological functions related to different agroecological principles ( Aubin *et al*., 2017). It is directly related to the third principle: using natural and local resources efficiently. However, it appears at the same time to be the practice restoring a high productivity level after water refilling. It allows the recycling of sediment material that accumulates during the production cycle to increase the concentration of mineral nutrients in the water for primary producers ( Aubin *et al*., 2017; Lierdeman, 2013). It should also allow for macrophytes re-establishment and control of algal blooms ( Aubin *et al*., 2017). As in temporary ponds, this voluntary dry period also promotes a very specific flora, especially macrophytes with a short life cycle ( Fontanilles *et al*., 2023).

Drying out a pond for one year affects both the physico-chemistry of the sediments and water but also the balance between primary producers and other biodiversity components such as macroinvertebrates. Theoretically, after the drying out, the aquatic plant community is composed of ruderal, fast-growing species, which allow the rapid recolonization of the environment. In subsequent years, the nutrient concentrations and the degree of pond eutrophication gradually increase. Ruderal plant species will be replaced by more competitive species ( Oertli & Frossard, 2013), and the ponds will subsequently switch into a turbid water state with low plant coverage and a dominance of phytoplankton in the water column. Earlier investigations show that such change in ecosystem state results in lower fish biomass production ( Horvath *et al*., 2002).

Although a periodic annual dry stand is an often applied management measure in fish ponds ( Arthaud *et al*., 2013; Aubin *et al*., 2017; Horvath *et al*., 2002; Lierdeman, 2013; Oertli & Frossard, 2013; Vanacker *et al*., 2016; Wezel *et al*., 2013), it is not yet well known to what extent such a dry period affects the functioning of the pond ecosystem with respect to physico-chemical characteristics, primary production, biodiversity, and fish production.

The present study therefore aimed to fill this important knowledge gap. More specifically, we aimed to analyse how a periodic one-year dry-out impacts the physico-chemical pond characteristics, the biomass and diversity of primary producers, invertebrate density and net fish yield. We hypothesized that (i) the dry-out enhances the mineralisation of organic matter accumulated in pond sediments and increases mineral nitrogen and phosphorus concentrations after water refilling, (ii) this mineral nutrient enrichment increases overall primary producers after the dry period, with a dominance of macrophytes over phytoplankton, and (iii) the diversity of macrophytes and benthic invertebrates’ densities are higher during the first year after the dry period and decreases over years in favour of a higher diversity and density of algae. Finally, (iv) drying out was expected to increase fish production with the highest net fish yield in the first year after drying out.

## Methods

### Study area and study sites

**Figure 1.**
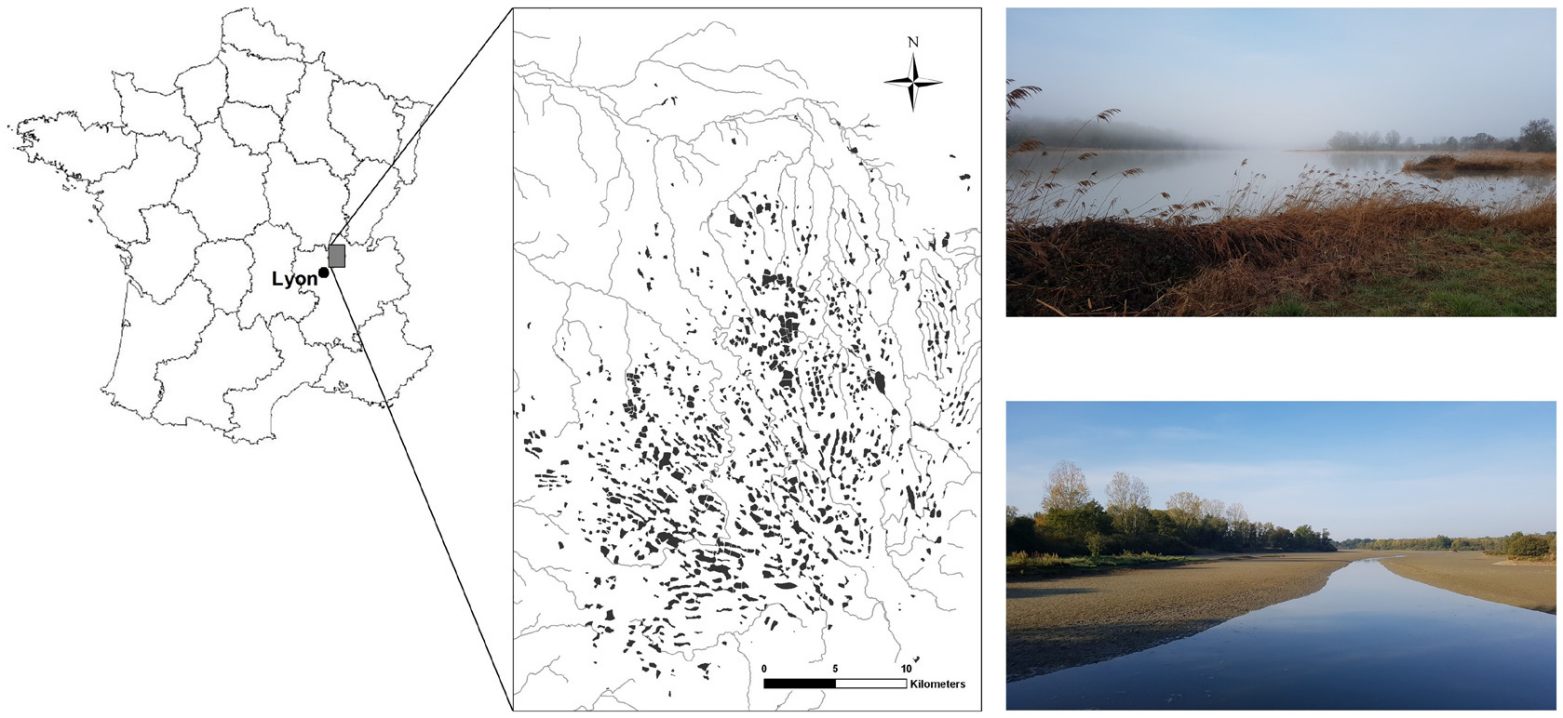
Location of the Dombes region in France (left) and its about 1,100 fish ponds (in black) and the river network (grey) (middle) ( Wezel *et al.*, 2013). Two pictures of a pond at the beginning of the production season (top-right) and a dry pond (down right).

The fish ponds studied were located in the Dombes area, in the department of Ain, Northeast of Lyon, in a Natura 2000 region ( Figure 1). This area is one of the main fish pond landscapes of France, comprising approximately 1,100 ponds and a total water surface of about 11,500 ha ( Bernard & Lebreton, 2007). These ponds also date back to the Middle Ages and have been used for extensive fish farming since then. They are organized in chains, with hydrological connectivity between ponds, according to annual draining for fish harvest. The ponds are therefore mainly filled with rainwater from ponds upstream in the chain. Historically, a mixture of common carp ( *Cyprinus carpio*), whitefish (roach and rudd), tench and piscivorous (pike and pikeperch) have been produced ( Wezel *et al*., 2013). Ponds are drained annually during the cold season (autumn and winter) to harvest the fish and are immediately refilled with water, in early spring. This fish production cycle typically lasts for four to five years, after which the ponds are left dry for an entire full growth season. This historical practice not only allows the mineralization of organic matter that has accumulated over the years of production, but also enables the manager to work on the infrastructure (dike, drainage system, pond profile, but also pathogen and invasive species management) if necessary. Historically, it also made the ponds available to farmers, who had additional land on which to grow crops. Average yield reaches 250 kg/ha ( Robin *et al*., 2014; Wezel *et al*., 2013), which corresponds to a rather low density of fish (45-70g/m ^3^) representative of extensive fish pond farming. The Dombes region is both an environment characterized by extensive fish farming with some ancestral practices but also by its biodiversity related to fish ponds ( Vallod & Wezel, 2010; Wezel *et al*., 2013; Wezel *et al*., 2014). The ponds monitored were selected with similar practices. Typical pond management practices include liming, fertilization and feeding fish.

The present study used a dataset holding key information (physico-chemical characteristics, biomass and diversity of primary producers, invertebrate density and net fish yield) from a set of 134-point data, including 85 different ponds that were monitored between 2007 and 2014, several years for some of them. These repetitions of some fish ponds in the dataset were considered in the analysis. Variable numbers of fish ponds represent each post-drying year from year to year ( Table 1).

**Table 1.** An overview of the number of fish ponds for each year class (Y1 correspond to the first year after drying out) and the different parameters that have been assessed.

Variables and years after dry out	Y1	Y2	Y3	Y4	Y>5
Organic matter concentration in sediments (g/kg)	31	35	31	22	15
Nitrate concentration in water (mg/l)					
Phosphate concentration in water (mg/l)					
Nitrate in total nitrogen in water (%)					
Chlorophyll a concentration (µg/l)					
Phytoplankton taxon richness	18	27	20	16	9
Cyanobacteria in phytoplankton (%)	11	20	16	12	9
Macrophytes cover (%)	29	29	28	21	15
Jackknife diversity of macrophytes	23	27	25	17	14
Density of invertebrates	16	11	6	7	7
Net fish yield (kg/ha/year)	26	28	24	17	10

These fish ponds are distinguished according to the number of years since the last dry out Y1 corresponds to the first year of water after the last drying out, Y2 the second year and so far, Y>5 includes all ponds that have been dry out five years ago or more ( Table 1). Expressed in a different way, this group includes fish ponds that have been in production for 5 years or more.

### Sediment physico-chemical parameters

We determined organic content and concentrations of nitrogen and phosphate in the sediment of each pond by collecting pond sediment twice a year (once in early spring [March] and once in autumn [October]) in each pond using an Eckman grab. Three samples were taken at different spots. Samples from different spots were homogenized into one sample in the field for later analysis in the laboratory.

The organic matter content of the sediment was determined in the laboratory according to the method ISO 10694 ( AFNOR, 1995). At the same time, we also determined the concentration of total nitrogen by dry combustion and exchangeable phosphorus following the method ISO 13878 ( AFNOR, 1998) and NF X31-160 ( AFNOR, 1999) respectively.

For organic matter, it was derived from the measurement of organic carbon, on which a conversion factor of 1.724 was applied (C/MO = 0.58), based on the method ISO 10694. The sample was heated to over 900°C to oxidize the carbon present into carbon dioxide. The amount of gas was measured. Data from spring and autumn were averaged for each pond prior to any statistical analysis.

### Water chemistry

Water samples were taken from each pond on 10 dates throughout spring and summer (from March to September). Water was collected at the deepest part of the pond, usually at the outlet, using a Van Dorn column to take a sample under the water surface. Samples were stored at 4°C until further analysis. Nitrate (NO ^3-^) and phosphate (PO _4_^2-^) were measured by ion chromatography (882 Compact IC plus, Metrohm) after filtration of the water samples using a syringe filter (RC CHROMAPHIL, 0.2 µm porosity). These concentrations are expressed in mg/l.

Total Nitrogen (TN) was measured on unfiltered water samples, following standard Hach® procedures (HACH Company, Loveland, Colorado, USA, DR/2400 Spectrophotometer Procedure Manual, Hach Company, 2002), with nitrogen persulfate reagent powder (410 nm wavelength, HACH method 10072). The method involves analysis by digestion, followed by absorbance reading by spectophotometry. The concentration is also expressed in mg/l and allowed to calculate the percentage of nitrate concentration on total nitrogen concentration in water.

### Phytoplankton

A subsample of the collected pond water was used to determine the concentration of chlorophyll a (CHL). For this purpose, a known volume of pond water was filtered through a Whatman GF/C filter which was subsequently incubated into a 90% acetone solution for 24 hours. After centrifugation, readings of absorbance were taken using a Shimadzu UV/VIS spectrophotometer UV-2101 (Schimadzu Corporation, Kyoto, Japan) at wavelengths of 630, 645, 663 and 750 nm. The CHL concentration was calculated based on the formula of Parsons and Strickland (1963). We used CHL concentration as a proxy for phytoplankton biomass.

In addition to this quantitative information, water samples also allowed to assess the phytoplankton community composition. The water was stored in a one-litre bottle and fixed with Lugol. Specimen identification was done to genus level in the laboratory using the Utermöhl method ( AFNOR, 2006). For each sample, 400 cells were counted under a Nikon Eclipse 50 microscope equipped with a Nikon DS-Fi1 camera. We obtained both the richness of the phytoplankton and the abundance of each genus.

### Macrophytes

The macrophyte community composition was assessed once in each pond during the growth season (between end June and July) by identification of specimens within multiple quadrats (4 m²) that were distributed every 50 meters along five transects following the protocol described in Arthaud *et al*. (2013) and after in Vanacker *et al*. ( 2015, 2016), and used since 2007. Transects were arranged perpendicularly to the pond’s dam, the deepest part with the outlet, of each pond. Two transects were located in the periphery, along the belt of helophytes, one in the center of the pond and the last two at half distance between the laterals and the central one. The number of quadrats was therefore related to the size of the pond. Macrophytes were identified to species level to obtain the richness per pond and their abundance within each quadrant was scored with the cover-abundance estimates of Braun-Blanquet (1932). This also allowed us to calculate the total coverage of macrophytes on the pond.

### Benthic invertebrates

Benthic invertebrates were sampled once in September in each pond every year. The protocol was based on the AFNOR NF T90-391 standard ( AFNOR, 2005) relating to the *“Indice Oligochètes de Bioindication Lacustre*” (IOBL). The oligochaetes present in the ponds live and feed in the sediments. These organisms play an important role in the functioning of the ecosystem, which is why this is an often monitored indicator. The standard protocol was supplemented by the technical note of Mazzella (2009) and by the experiences in the collection in these types of ponds in the Dombes area ( Vallod *et al*., 2011).

In the same way as mentioned above, the sediments were collected using an Eckman grab. Two samples were taken in an area of maximum depth reachable by foot and two others in shallower depth. These four samples allowed the equivalent of 0.1 m ^2^ of sediment to be collected. Each point had to be at least 10 m away from the others. They were carried out in the sector of the outlet without getting too close to it in order to be representative of the environmental conditions. These samples were pooled to form more than one sample for analysis.

The samples were first filtered in the field using a 0.315 mm sieve and the residue was then fixed with 5% formalin. This sample was then processed in the laboratory to determine the density of oligochaetes, corresponding to a number of individuals per area. It was measured using the formula: 3.log(1+(100.EFF)) where 100.EFF is the number of individuals per area per 0.1 m ^2^.

### Fish

Information on the net fish yield was obtained from local fish farmers and collected by the local fish association. It corresponds to the total fish biomass harvested at the end of the season (Autumn) minus the total stocked fish biomass at the start of the growth season (early Spring). Fish yields are expressed in kg/ha/year.

### Statistical analysis

All statistical analyses were conducted with R and R Studio software ( R Development Core Team, 2013).

For macrophytes richness, we used the Jackknife index based on Vanacker (2016). It allows to obtain a better estimation of the richness, often underestimated in fish ponds. Vanacker showed that it was the most suitable index for these environments and their biodiversity ( Vanacker, 2016), this has also been shown more generally by Soukainen and Cardoso (2022). The Jackknife index appears to be the most accurate, efficient and least biased of the non-parametric indices for estimating species richness ( Vanacker, 2016). It is calculated based on the number of species returned once and the number of quadrats made.

The values of water parameters used were the medians of the various measurements taken during the year (April to July), to have a better representation, as the mean is more driven by extreme values.

Firstly, to observe the variations of the different variables studied over the years since the last drying out, we created boxplots using functions from the ggplot2 package ( Wickham, 2016).

We used Levene tests to compare the variability of the variables between ponds monitored several years and ponds monitored only once. No significant differences were found. These results are in line with those highlighted by Vanacker *et al*. (2016). One reason for this is that the ponds are emptied every year. But to take repetitions of some fish ponds into account, we also performed linear mixed models with the glmer function of the lme4 package ( Bates, 2010). Depending on the variables, we used Poisson, negative binomial or Gaussian laws. The models used and their p-values are shown alongside the boxplots. This makes it possible to show if there is an effect of the practice of drying out on the variables studied.

The models are in the form of (DryOut represents the year after the last drying out and PondID the identifier of each pond to take into account repetitions):


Variable studied ∼ DryOut + (1|PondID)

Then, to analyze the relationships between variables, we looked for correlations of Spearman, using the ggcormat function of the ggstatsplot package ( Patil, 2021). The graphical representations are made with the scatterplot function of the car package ( Fox & Weisberg, 2019).

Finally, in order to obtain a synthetic and multivariate vision of the variations observed with the boxplots, we performed a multivariate analysis with a Principal Component Analysis (PCA). This ordination has been made with the dudi.pca function from the ade4 package ( Thioulouse *et al*., 2018). The functions s.class, s.arrow and superpose from the same package allow for graphical representations.

## Results

134-point data, including 85 different fish ponds have been monitored between 2007 and 2014. For all variables, we observed a large variability between fish ponds ( Table 2). This high variability was similar both between different ponds and within the same pond over several years. There was both variability in physico-chemical parameters but also in biological parameters.

**Table 2.** The median, minimum and maximum values for the parameters studied.

Variables	Median	Min	Max
Organic matter concentration in sediments (g/kg)	33.69	16.58	112.41
Nitrate concentration in water (mg/l NO ^3-^)	0.47	<0.025	1
Phosphate concentration in water (mg/l PO _2_^4-^)	0.089	0.007	0.5812
Nitrate in total nitrogen in water (%)	19	0	82
Chlorophyll a concentration (µg/l)	48.06	2.92	347.51
Phytoplankton taxon richness	32	16	45
Cyanobacteria in phytoplankton (% of biomass)	23	0	71
Macrophytes cover (%)	34	0	144
Jackknife diversity of macrophytes	14.9	0	43.8
Density of invertebrates	1670	22	4764
Net fish yield (kg/ha/year)	185	8	696

Sediment composition varied between ponds and similarly, so did nutrient concentrations in the water. For example, we observed a concentration of organic matter in the sediment ranging from 16.8 g/kg to over 110 g/kg. Nitrate concentration in the water ranged from near zero to 1 mg/L.

Similarly, ponds differed strongly in local macrophyte species richness: some ponds had no macrophyte species while others had more than 40. In the whole set of ponds, 96 species of macrophytes were sampled. Some species were very common and are found in more than 50% of the ponds such as *Polygonum amphibium*, *Najas minor* and *Najas marina*, *Phalaris arundimacea*, *Potamogeton nodosus* and *Potamogeton crispus*. Conversely, some species were present in only one. Some ponds also hosted species threatened at the French or European level like *Elatine alsinastrum*, *E. hexandra or E. hydropiper*, *Hydrocharis morus ranae*, *Luronium natans*, and *Marsilea quadrifolia*.

Finally, net fish yields varied from a few kilos per hectare to almost 700 kg/ha/year.

### Physico-chemistry of sediments and water

Higher water nutrient concentrations, especially for nitrate, could be found during Y1 and Y2, and then decreasing in the following years (p<0.05 for glmer). We observed a median concentration of nitrate of 0.47 mg/L in Y1 and 0.52 mg/L in Y2 against 0.36 mg/L in Y4. Although the different pond categories did not differ significantly in phosphate concentrations, we observed a similar trend with lower values with increasing years after drying out. The highest concentration was measured in Y1 ponds (median of 0.12 mg/L) and the lowest concentrations were observed in Y4 ponds (median of 0.07 mg/L).

**Figure 2.**
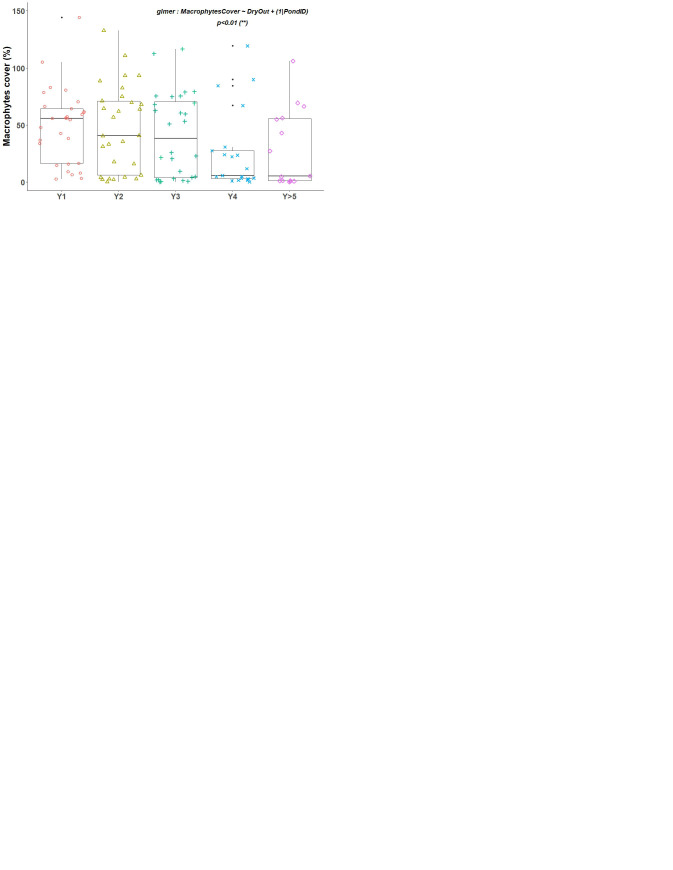
Variation of different parameters monitored in fish ponds of the Dombes area, France, according to the years after being drying out for one year. Each point corresponds to a fish pond monitored over one year. The associated and tested glmer model is inserted on each graph, along with the associated p-value.

More specifically, the percentage of nitrate on total nitrogen decreases significantly (p<0.05 for glmer) from Y1 to Y4 ( Figure 2). It reduced by a factor 3 over a period of four years (from 30% the first year to 10% the fourth).

For organic matter concentration in sediments, no accumulation was observed from Y1 to Y5 ( Figure 2). A slight, but not significant increase of less than 1 g/kg of sediment was present over the first three years (Y1 to Y3). The trend observed was even rather downward, with less organic matter over the years. Results were similar for nitrate with no significant difference between year classes, as well as for available phosphorus, even if a slight increase for this was observed between classes Y1 and Y>5.

### Quantitative parameters for primary producers

The maximum of macrophytes cover (median value) was found in Y1, before a gradual decrease ( Figure 2, p<0.01 for glmer). On average 56% of the pond surface was covered by macrophytes in Y1. It decreased to around 40% in Y2 and Y3, and then collapsed to 5% in Y4. In the following years it remained at this low coverage.

Conversely, algal concentration (CHL) was low in Y1 with 30 µg/L and increased in the following years ( Figure 2). The mixed model tested showed an effect of water year on this algal concentration (p<0.001). The maximum was reached during Y4 with an average concentration around 58 µg/L. However, contrary to what can be expected, the increase of the CHL concentration was not continuous, notably after 6 years. After 10–15 years, the primary producers’ biomass in the ponds becomes low.

### Primary producers diversity and invertebrates density

**Figure 3.**
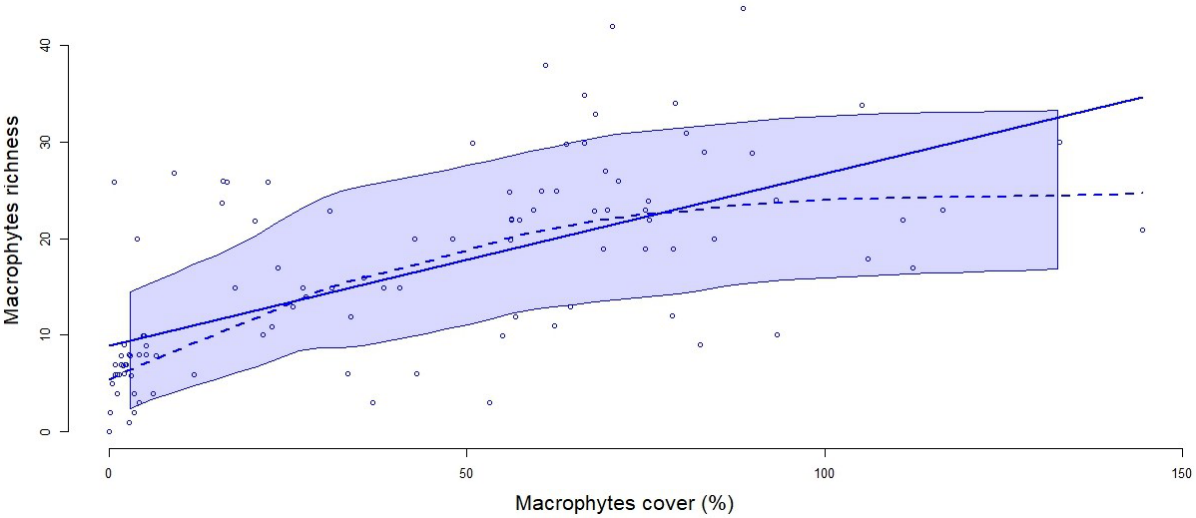
Correlation between macrophyte richness (with a Jackknife index) and cover (expressed in %). The solid line represents the linear regression between the two variables (R ^2^=0.732, p<0.0001) and the dashed line represents a smoothed non-parametric regression. The band around the curves represent the confident interval with a level of 0.95.

According to the index of Jackknife, macrophyte richness declined continuously other the years (p<0.01 for glmer), with a median number of 23 species in Y1 and 8 in Y5 and after. This richness of macrophytes was positively and significantly correlated with cover extent ( Figure 3).

**Figure 4.**
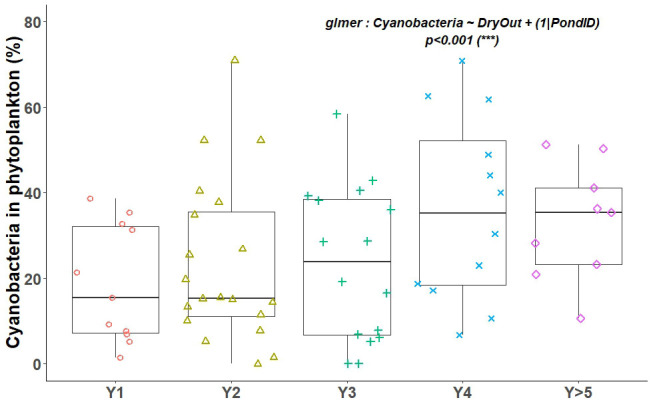
Percentage of cyanobacteria in phytoplankton as a function of year after drying out. Each point corresponds to a fish pond monitored over one year. The associated and tested glmer model is inserted on each graph, along with the associated p-value.

**Figure 5.**
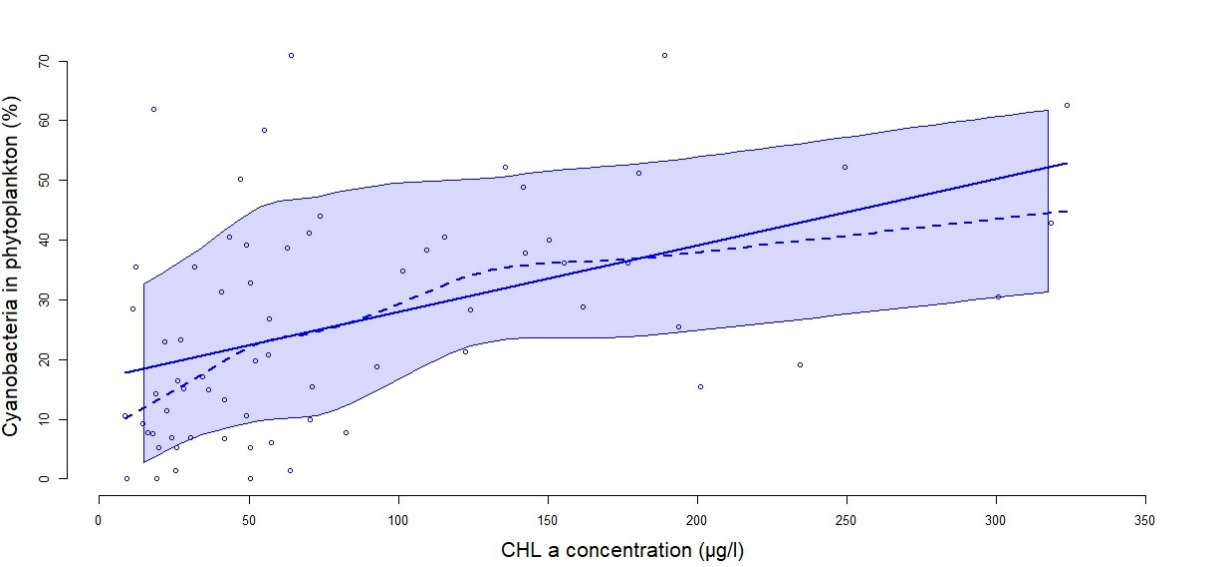
Correlation between the percentage of cyanobacteria and CHL concentration (expressed in µg/l). The solid line represents the linear regression between the two variables (R ^2^=0.47, p<0.0001) and the dashed line represents a smoothed non-parametric regression. The band around the curves represent the confident interval with a level of 0.95.

For phytoplankton, we did not have the same positive correlation between concentration and richness. The richness was higher when the CHL concentrations are low ( Figure 2). It reached its maximum after five years with water, with a median of 36 taxa, and was also high in Y1 with 35 taxa. Lower values were observed in the years Y2 to Y4. Over the years, the relative abundance of cyanobacteria among phytoplankton increased gradually ( Figure 4). The mixed model tested showed an effect of years after drying out (p<0.001). A positive correlation between CHL concentration and cyanobacteria abundance was found. When CHL increased above 100 µg/L, this relative abundance exceeded 20% ( Figure 5).

Specifically for invertebrates, even if densities were lower in Y1 than in Y>5 (medians of 397 *versus* 1,594 individuals), they were higher in Y2 and Y4 than in Y>5 ( Figure 2). The densities were at a maximum in Y4 with 2,579 individuals, followed closely by the second year with 2,286 individuals. The mixed model did not show a significant relationship.

In summary, macrophytes richness and phytoplankton richness were higher in Y1, and in Y2 for invertebrates densities ( Figure 2).

### Fish yield

**Figure 6.**
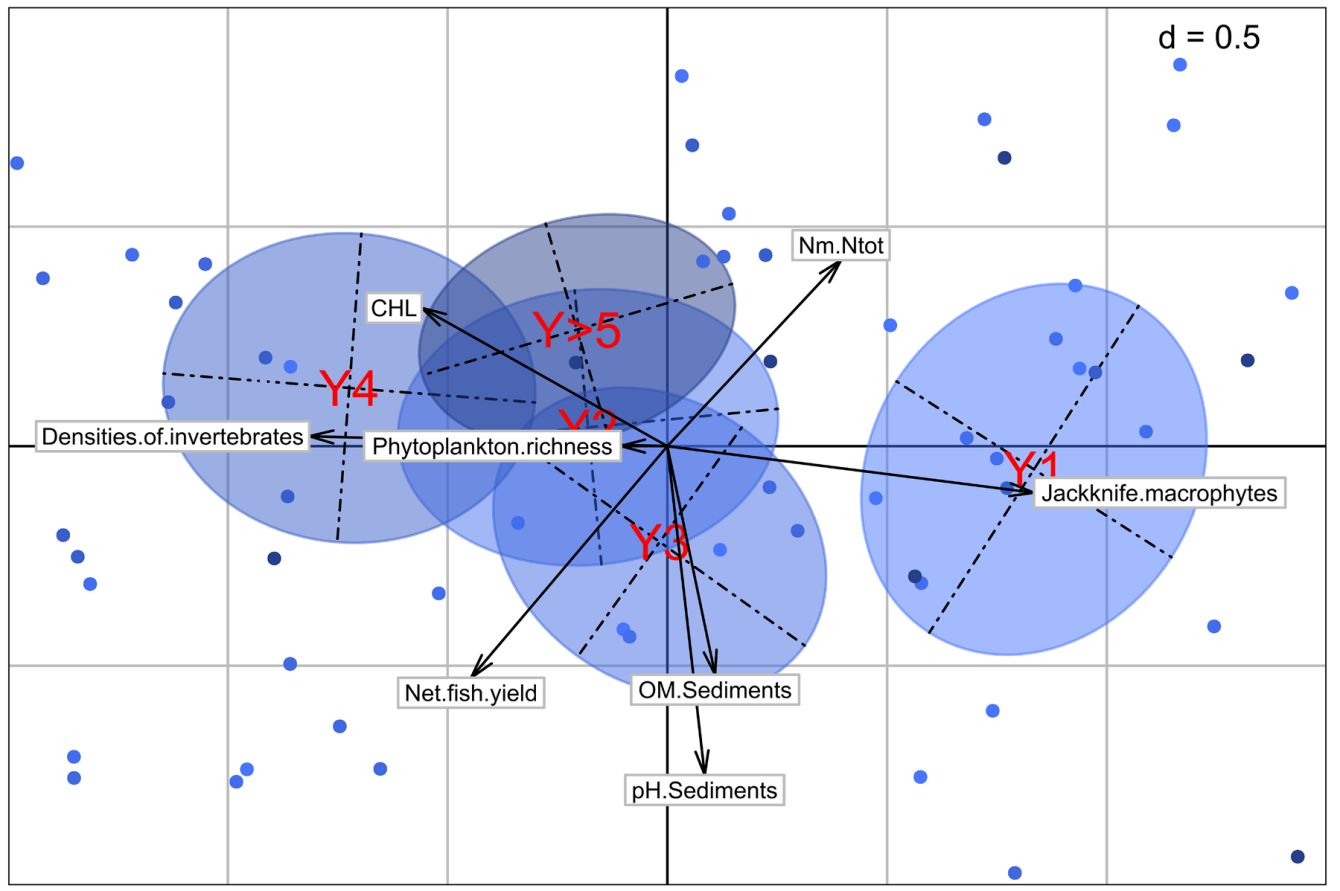
Positioning of years after the last dry out and variables on the factorial map. The points correspond to the individuals, i.e. the fish ponds, the arrows and their length to the correlation of the variables with the first two dimensions of the PCA, the ellipses represent a graphical summary of the scatterplots corresponding to each pond class. The center of the ellipse is the center of gravity.

The highest fish yields were achieved in Y1 and Y2 (196 kg/ha and 214 kg/ha respectively) compared to 152 kg/ha for Y>5 ponds ( Figure 2). A strong link between this yield and invertebrate density was observed (Correlation R ^2^=0.41, p<0.05, and multivariate analyses, Figure 6). p<0.05 for the mixed model tested.

### Multivariate analysis

The multivariate analysis with a PCA provides the position of the five year classes of our dataset regarding different variables measured ( Figure 6). The trajectory is mainly observed on the first dimension of the analysis, starting from the positive part of the X-axis (in the right part) with Y1 and going to the negative part (in the left) with Y4. Ponds that have been in water for 5 years or more are more located towards the center of the plot. In terms of variables, this trajectory corresponds to a shift from an agroecosystem dominated by macrophytes to one dominated by algae. A progressive increase in invertebrate density with the sawtooth evolution can be also observed as before in the analysis. The positioning of the ponds with water for more than 5 years shows a decrease of the primary productivity as a whole, with less good fish yields.

In an addition analysis where Y>5 ponds are separated for the respective exact years, we see the same results for the first four years classes. The fifth year seems to follow a similar logic with still good invertebrate densities but, the end of this evolution occurs with the sixth year, where the primary production decreases in an overall way. The positioning of the following years seems more random, with a logic more difficult to define and explain.

## Discussion

The aim of this study was to analyse the effects of the practice of drying out on the ecological functioning of a fish pond, on its biodiversity but also on its fish productivity. Our results showed that this practice leads to mineralization of organic matter, with a higher concentration of inorganic nitrogen and phosphorus in the water in the first year after being dry out. It also allows macrophytes to recolonize the environment, before phytoplankton gradually dominates in subsequent years. In particular, the second year is marked by a balance between the two primary producers, associated with greater biodiversity and better fish yields. All these elements show that drying out ponds for several months or a year is a major agroecological practice for reconciling production and biodiversity conservation, and that its frequency of application should be reconsidered.

### Accumulation of organic matter and mineral nutrients in fish ponds

The accumulation of organic matter in the bottom of the pond is a natural process in all standing water bodies, called sedimentation ( Oertli & Frossard, 2013). In the context of the Dombes fish ponds, we analysed the concentration of organic matter in the sediments according to the years after the last dry-out of the ponds. We expected an increase in organic matter content over the years; however, our results based on means for each year after drying did not support this, and thus did not confirming our hypothesis. The organic matter content in the sediments remained relatively stable for all the years studied, and no significant differences could be found. The fish farming practice based on emptying the pond every year in order to harvest fish in a small remaining part of the pond may explain these observations. During this, the more labile parts of the sediments can be washed away from the pond with the water drainage at the pond outlet. Vallod and Sarrazin (2010) showed that for a 24-ha pond, 8.5 t of material were exported during emptying. Moreover, the period during draining when the sediments in the largest part of the ponds remain in contact with air, even if it is much shorter than during a one-year dry period, probably already allows a partial mineralisation of the organic matter that has accumulated, if the temperatures and oxygen concentrations are favourable for microbial activity ( Boyd & Pippopinyo, 1994; Boyd *et al*., 2002). Indeed, the pond is drained between September and January and water refilling can take several weeks, depending on rainfall events and water transport from surrounding land areas via water ditches toward the ponds.

Our results show higher concentrations of inorganic nitrogen and phosphorus in the water after the dry-out years Y1 and Y2. Despite this, we did not observe any accumulation of organic matter in the sediments. We can nevertheless make the hypothesis that the drying up favours an incomplete mineralization of the organic matter in the sediments. Draining of the ponds increases the oxygen concentration in the sediments and thus promote the decomposition of organic matter ( Boyd *et al*., 2002). These results confirm the interest of drying out ponds for nutrients mineralization and availability in the water column ( Avocat, 1975; Billard, 1979; Lierdeman, 2013; Wezel *et al*., 2013).

### Primary production and competition between algae and macrophytes

In ponds, two types of primary producers, algae and macrophytes, compete for light and nutrients. Scheffer and Carpenter (2003) established the theory of alternative stable states, with the relative domination of one or another. Our results show a domination of macrophytes in Y1, as shown by their high percent coverage. These results agree with those of Vanacker *et al*. (2016), who observed an average pond coverage of macrophytes in Y1 close to our results (49% versus 56% here). These macrophytes are a good indicator of biodiversity and productivity of the aquatic ecosystem ( Folke *et al*., 2004). In the following years, we observed a progressive decrease, which likely resulted from a gradual increase in water turbidity caused by enhanced concentrations of phytoplankton. After the drying period, a first stage appeared during Y1 with a domination of macrophytes and a progressive shift between primary producers in Y2 and Y3. In Y4 and Y>5, we noted the domination of phytoplankton and a collapse of macrophyte cover and richness, thus validating our initial hypothesis. However, if we look at the following years included in Y>5, the concentration of both primary producers decreased thus reaching a third stage where the two primary producers were in low biomass quantity. This would suggest a decrease of the total primary production. Finally, after a dominance of macrophytes in Y1 and a progressive shift (Y2 and Y3) towards phytoplankton dominance (Y4), ponds could evolve to a detrital state based on decomposers ( Okuda *et al*., 2013).

In fish ponds, we would ideally maintain a continuous balance between these two primary producers in order to ensure a good productivity of the agroecosystem. This could correspond to an unstable state, between the two stable states defined by Scheffer and Carpenter (2003). Our results confirm that this situation of a balance between macrophytes and phytoplankton is mostly observed in Y2, but changes afterwards. Therefore, this balance is of interest for fish production systems to ensure a high productivity in the food web. In addition, the presence of a balance between both primary producers ensures that linked biodiversity is maintained. In our study, during Y2, macrophyte coverage averaged 40% of the pond surface and CHL concentration was around 53 µg/L. Regarding macrophytes, our results are higher than those recommended by Schlumberger and Girard (2020), which were 15 to 20% macrophyte cover in extensive fish ponds. For phytoplankton, our results were below 60µg/L of CHL defined by Vanacker *et al*. (2015) as a tipping point above which they observed a significant decrease in aquatic plant diversity. Thus, the dry season allows for the rejuvenation of the agroecosystem, but also for the initiation of a succession, especially for macrophytes ( Arthaud *et al*., 2013). Here, Y2 were characterized by a high richness and abundance of macrophytes, by a balance between plant and phytoplankton, and simultaneously by the highest fish yields. A complementary study with zooplankton monitoring could be interesting to demonstrate that Y2 proposed the highest productivity at the different links of the food web.

### Effects of dry year disturbance on biodiversity

The diversity of macrophytes was found to be directly correlated with the macrophytes cover of the ponds. The macrophyte species richness was at its maximum in Y1 and then decreased in the following years. During Y1, we found an average of more than 22 species while then decreasing to about 16 in Y2 and only 11 in Y4. On average, fish ponds in the Dombes area host between 11 and 15 aquatic plant species ( Arthaud *et al*., 2013; Robin *et al*., 2014; Vanacker *et al*., 2015; Wezel *et al*., 2013). Our analysis shows that the macrophyte richness was different for each year, higher in Y1 and progressively decreased over the years.

In this paper, we were interested in the biodiversity of macrophytes only through the number of species. For biodiversity conservation purposes, it is also interesting to analyse the composition of the communities and their variation in different years. In a study on this, Fontanilles *et al*. (2023) highlighted about fifteen species specific to Y1. These are species with a high recolonization capacity or that are more tolerant to disturbance. Some species need this alternation between a dry phase and a wet phase. We found rare species, disappearing the following years. For the others years after drying-out, they observed the phenomenon of nestedness based on a loss of several species but not on a complete turnover ( Fontanilles *et al*., 2023).

Related to richness of phytoplankton taxa and CHL concentration, we found a negative correlation. These results are consistent with those found by Qin *et al*. (2013), showing that eutrophication decreases phytoplankton diversity. Phytoplankton richness was, like macrophytes, at its maximum during Y1 and then comparatively low in Y>5 ponds. We also observed that when the concentration of CHL increased, the percentage of cyanobacteria also increased to the detriment of other types of algae, resulting in an overall loss of phytoplankton taxa diversity. It is known that when phytoplankton concentration increases during eutrophication, the biomass of cyanobacteria increases and leads to changes in taxonomic structure ( Havens, 2008). Our results are therefore similar. This proliferation of cyanobacteria should be avoided because it leads to both a loss of biodiversity and a significant decrease in oxygen in the water. This anoxia can lead in some cases to strong fish losses.

For invertebrates, the density was not at its maximum in Y1 but in Y2 and Y4. The recolonization time of these taxa appears to be longer after the disturbance of dry-out. Here we have only quantitative information with the density of invertebrates but not on the diversity present. Few studies have addressed the effect of drying out a pond on aquatic invertebrates. Sayer *et al*. (2012) showed that diversity was higher in managed ponds. The management practices included the removal of some of the sediments and trees growing around the pond. They can be compared to a dry-out with the objective of rejuvenating the agroecosystem. The duration of the dry period also appears to be an important factor in the ability to recolonize. Boix *et al*. (2001) presented an example with three pioneer species, present after six months of drying out but disappearing after two years. Further studies on invertebrate diversity should be conducted to confirm that invertebrate communities follow the same evolution cycle between two drying out from Y1 to Y>5, as phytoplankton or macrophytes.

### Drying out a pond as an important agroecological practice in extensive fish farming

Drying out ponds allows a mineralisation process, which ensures a greater availability of nutrients in the water during Y1. This allows a good development and a balance between the primary producers. This practice also promotes higher levels of macrophyte richness in the ponds and a higher density of invertebrates, a major source of food for the fish, especially in Y2. All these elements explain the better fish yields observed, especially in the second year of production. They also validate the hypothesis that drying out ponds appears as an important agroecological practice in extensive fish farming.

**Figure 7.**
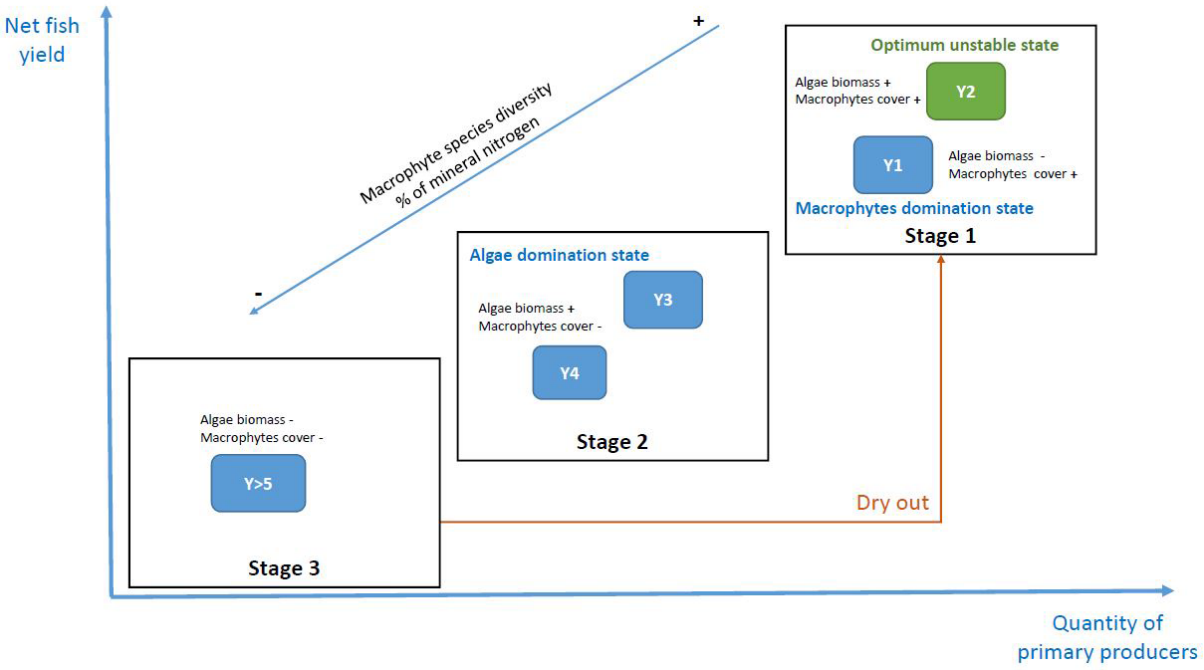
Conceptual model of the three stages identified in the temporal changes of a fish pond in the Dombes area, France. The x-axis represents primary productivity (algae and macrophytes) and the y-axis represents fish productivity. Year 2 (Y2) was the one with the best balance in a fish production objective. The percentage of mineral nitrogen is in water.

Our results are in line with some other research such as Vanacker *et al*. (2016) who stated that it is essential to dry out regularly to ensure good fish yields, as well as those of Horvath *et al*. (2002) who also confirmed the beneficial role of a period of drying out. We can see that, in addition to fish production, it is the entire functioning of the agroecosystem, based on primary production but also some species or taxa diversity, that benefits from this practice, in certain periods after the pond drying out. The cycle of different states, water and sediment quality, fish yield, and elements of biodiversity is illustrated in Figure 7. The drying out of ponds promotes a higher productivity of the agroecosystem during two or three years. The organic matter in the sediments undergoes mineralization during the dry period, making nitrate and phosphate available in the water the following years for primary production and thus also provides resources for fish. This enhanced mineralization can also limit the use of inputs during the fish production cycle. The disturbance through drying out and the available nutrients thereafter seem to support the recolonization of macrophytes. Indeed, in Y2, a good state of equilibrium between the two types of primary producers is observed, but also macroinvertebrates in higher densities, altogether allowing better fish yields ( Figure 7). These observations are directly linked to one of the principles of agroecological fish pond management: “an agroecological fish pond is robust and resilient” ( Aubin *et al*., 2017). Moreover, drying out allows also to reach higher levels of aquatic plant and algal diversity, at least in the first few years. These refer also to the other principles such as being environmentally friendly (principle 4), and having a natural and cultural value (5). The drying out also indicates a certain resilience and robustness of the ecosystem (2), which is however mostly pronounced in Y1 and Y2.

Our results might also justify the will of local actors in the study area to join the RAMSAR convention on wetlands of international importance, which rewards sites and actors who want to preserve wetlands. The role of fish farmers applying the production in an agroecological way is of first importance to meet the objectives of biodiversity conservation. In typical European pondscapes where fish production remains, maintaining this activity is essential for pond subsistence.

### Frequency of drying out fish ponds

All the variables studied do not react in the same way to the disturbance caused by drying out ponds and do not evolve in the same way in the following years. Therefore, the question of the best dry-out frequency is important for the management of ponds from a production, but also ecological perspective. Horvath *et al*. (2002) mentioned that a dry period should be carried out regularly, but did not provide any details, whereas Sayer *et al*. (2012) stated that three to five years after sediment removal (ponds were dried out and sediments removed with mechanic action), macrophyte and invertebrate biodiversity decreased significantly. This is similar to findings by Vanacker *et al*. (2016) on determining tipping points in fish pond systems, who highlighted a significant decline in their performance indicator after Y>5. This indicator was based on aquatic plant richness, CHL, and fish yield. They stated that ideally, a pond should have more than 15 species of macrophytes, a CHL concentration below 60 µg/L – tthe threshold above which tipping points of different environmental variables are observed – and may assure a fish yield above 213 kg/ha. Our results show that we had a richness of macrophytes below 15 from four years onwards. The tipping point of 60 µg/L of CHL was not reached, but was very close during Y4. Finally, a fish yield of 213 kg/ha was only reached in the second year, but was lower in all other years. These median values were very close to the averages stated in the 2021 report of the local fish promotion association.

Our results suggest that ponds should be dried out every three years. Indeed, ecosystem productivity, biodiversity levels and fish yields were at their maximum in the second year and then declined. Drying out at the end of the third year would allow a rapid return to these more favourable levels. Historically, the cycle has been of five or six years. Reducing it to four years would also have repercussions on water distribution. Indeed, each year a quarter of the ponds would be dry, instead of the current one-fifth. In a context of climate change and water shortage, this could be a solution for a better filling of the ponds.

Only very few ponds in our study area are kept in water for more than five years. It would be interesting to analyse the effects of this practice in other fish farming regions. Indeed, effects may differ between such a drying out practice done for the first time and conducted historically, as water and biodiversity variables might potentially vary considerably as disturbance regimes are different. Another important point to emphasize concerns the variability observed on all the parameters studied in this paper. Indeed, even if the practice of drying out ponds, for an entire growth season, is a factor explaining interannual variability, the latter was highly significant in each class. It would be interesting to investigate which factors explain this variability.

## Conclusions

Fish ponds face a double challenge: to produce fish to ensure a sufficient income for pond managers while conserving biodiversity. Indeed, some pondscapes are considered as sites for biodiversity conservation such as a Natura 2000 zones or Ramsar sites, as discussed in the study area by local and regional stakeholders for the past few years. Agroecological practices must reconcile the two, through supporting an optimized functioning of the agroecosystem. Among all these practices, drying out ponds, although a major disturbance, allow a good functioning of this agroecosystem, permitting fish production while at the same time conserving biodiversity.

Our results do not show an accumulation of organic matter in the sediments, as we predicted. Nevertheless, our results suggest that a dry year allows the mineralization of nitrogen in pond sediments and enhances the availability of mineral nitrogen in the water column during the first year after dry stand (Y1). In connection with these nutrient dynamics, this practice allows the restoration of a good primary productivity and a significant colonization by macrophytes. Indeed, it favours a domination of the agroecosystem by macrophytes with a low phytoplankton development in Y1. Drying out resets the systems towards a macrophyte dominate state. In connection with this, we also observed a higher specific diversity.

The second year (Y2) marked the best state of balance between the two primary producers, an optimum unstable state for fish production. It was also the year with the highest density of invertebrates. These different elements explain why it was also the best year for fish yields.

During the following years (Y3 and Y4), we observed a decrease in inorganic nutrients availability in the water, as well as in the cover and the species diversity of macrophytes, in favour of the quantity of phytoplankton.

For ponds maintained in water for a longer period (more than five years), we observed a lower presence of the two primary producers: a low cover of macrophytes and a lower concentration of phytoplankton. The same was true for their diversity, which was at lower levels than in the first years. This change of functioning is beneficial for a detrivorous food web, also called microbial loop. The use of a dry period in these situations allows the rejuvenation of the ecosystem with a major disturbance. As we have seen, this allows a return to good levels of primary productivity and therefore fish production, while maintaining the highest levels of biodiversity.

Drying ponds out for one year, or at least for some months, might also be a practice to be implemented regularly in some natural ponds where organic matter has accumulated, more than sediment dredging techniques which are expensive and often inefficient on a long-term scale. This practice could also be extended to other types of ponds, such as irrigation ponds or urban ponds, to promote biodiversity and avoid future pond filling.

Future research on agroecology-based management of water systems could be enlarged to other agroecological practices that rely on diversification. This could include testing and evaluating on having a larger diversity of fish species and that could provide enhanced ecosystem services such as provision service with more stable fish yields, enhanced but sustainable used of natural resources such as phyto- and zooplankton as feed, and also resilience of the overall fish production systems under more erratic climatic conditions to be expected with climate change.

## Data Availability

Zenodo: Dataset - Drying out fish ponds, for an entire growth season, as an agroecological practice: maintaining primary producers for fish production and biodiversity conservation, https://doi.org/10.5281/zenodo.8183091 ( Girard *et al*., 2023) This project contains the following underlying data: - Girard *et al.* - 2023 - Dataset.csv - Girard *et al.* - 2023 - Metadata.csv Data are available under the terms of the Creative Commons Zero "No rights reserved" data waiver (CC0 1.0 Public domain dedication).
