# Supplementary material for: Drying out fish ponds, for an entire growth season, as an agroecological practice: maintaining primary producers for fish production and biodiversity conservation
Source: Open Res Eur. 2024 Oct 15;3:125. Originally published 2023 Aug 18. [Version 2] doi: 10.12688/openreseurope.16363.2 (PMC11519614; doi:10.12688/openreseurope.16363.2)
Supplement: Peer review history

# Reviewer report: Reviewer response for version 2

**DOI:** 10.21956/openreseurope.20142.r45196

**Published:** 2024-11-09

**Authors:** Koushik Roy

**Affiliations:** 1 Institute of Aquaculture and Protection of Waters, Faculty of Fisheries and Protection of Waters, University of South Bohemia in Ceske Budejovice, Ceske Budejovice, Czech Republic

1. The additional aspects surrounding the agro-ecological practice of drying-out pond needs to be presented in the MS: "supporting measures such as draining, partial cultivation of the pond bowl, sowing, etc., were practiced? Advantage not only limited to enhancing aerobic processes but allows killing of potential pathogens too. This was not discussed?

 2. Regular muddy sediments of 10–15 cm in height are regarded as adequate. Pond mud usually accumulates in the deepest part of pond, which often is the fish

 harvesting ground. Was there any such threshold in the agro-ecological process decision making? such as to dry or not to dry decision... what are authors observation/ recommendation?

 3. The authors did not discuss common practices with references from other parts of Europe, where European fishponds are a major aquaculture system unlike in France. Such literature should be discussed. For example, in Central Eastern Europe, wintering the exposed pond bed should be done at least once in 4 years. Whereas summering of exposed pond-bed is recommended at least once in 10-14 years. Read Hartman et al. 2015 ("Carp Aquaculture in Europe and Asia").[Ref 1] 

 4. The paper is valuable. But missing several important agronomical data on soil/ sediment. Especially I miss the bulk density of sediment (expressed in g dry weight/cm3) before and after drying. Bulk density trends show carbon sequestration by water bodies Boyd CE et al, 2010 [Ref 2]  ; Nag S et al, 2019 [Ref 3] . This could have been a valuable addition to the study.

 5. Alao it is a pity that only water quality data was measured and not sediment (given that drying is focused on sediment quality), such as C, N, P content on dry matter basis, Including changes in C:N, N:P, and C:P molar ratios - before and after drying or year-to-year progression after drying. If you have data, please provide it. If not, please discuss it as a limitation of the study, and encourage future directions.

 6. Stoichiometric data or data useful for understanding ecological stoichiometry changes "before and after" or at least "year by year" after implementing the agro-ecological practice is not given. Please compute water DIN: TP ratio (molar) of water column before and after drying or progression by year after drying. Comment on potential resetting of N and P limitations for the phytoplankton at DIN:TP < 2.0 and > 5.1, respectively. Adopt it from: Vrba J et al, 2023 [Ref 4] 

 7. Comment how your proposed agro-ecological practice would enable pond aquaculture to operate within the water framework directive quite comfortably. By commenting on the changes in Upper permissible limits of PO4 (0.4 mg L−1) or and NH _4_ (1 mg L ^−1^)  according to EU Directive 2006/44/E Article 3 & 5, Annex I - before and after drying or year-to-year progression after drying.

 8. Do you have some data/ unpublished data (previous) on any long-time non-dried pond(s)? that could be visualized as control to make it more contrasting.

Is the study design appropriate and does the work have academic merit?

Partly

Is the work clearly and accurately presented and does it cite the current literature?

Yes

If applicable, is the statistical analysis and its interpretation appropriate?

Yes

Are all the source data underlying the results available to ensure full reproducibility?

Yes

Are the conclusions drawn adequately supported by the results?

Partly

Are sufficient details of methods and analysis provided to allow replication by others?

Yes

Reviewer Expertise:

Fish nutrition, Freshwater ecology, Fishponds.

I confirm that I have read this submission and believe that I have an appropriate level of expertise to confirm that it is of an acceptable scientific standard, however I have significant reservations, as outlined above.

## References

[1] The Natural History of Common Carp and Common Carp Genetics.2015; 10.1201/b18547-6 17-40 10.1201/b18547-6

[2] : Role of aquaculture pond sediments in sequestration of annual global carbon emissions. *Environ Pollut* .2010;158(8) : 10.1016/j.envpol.2010.04.025 2537-40 10.1016/j.envpol.2010.04.025 20537775

[3] : Carbon balance of a sewage-fed aquaculture wetland. *Wetlands Ecology and Management* .2019;27(2-3) : 10.1007/s11273-019-09661-8 311-322 10.1007/s11273-019-09661-8

[4] : Top-down and bottom-up control of plankton structure and dynamics in hypertrophic fishponds. *Hydrobiologia* .2023; 10.1007/s10750-023-05312-5 10.1007/s10750-023-05312-5

# Reviewer report: Reviewer response for version 2

**DOI:** 10.21956/openreseurope.20142.r45173

**Published:** 2024-10-28

**Authors:** Eric Fouilland

**Affiliations:** 1 Centre for Marine Biodiversity Exploitation and Conservation, Montpellier, Occitanie, France

I have reviewed the revised manuscript and the responses to my comments, and I change the status of my review to Approved.

Is the study design appropriate and does the work have academic merit?

Yes

Is the work clearly and accurately presented and does it cite the current literature?

Yes

If applicable, is the statistical analysis and its interpretation appropriate?

Partly

Are all the source data underlying the results available to ensure full reproducibility?

Partly

Are the conclusions drawn adequately supported by the results?

Partly

Are sufficient details of methods and analysis provided to allow replication by others?

Partly

Reviewer Expertise:

microbial ecology

I confirm that I have read this submission and believe that I have an appropriate level of expertise to confirm that it is of an acceptable scientific standard.

# Reviewer report: Reviewer response for version 2

**DOI:** 10.21956/openreseurope.20142.r45172

**Published:** 2024-10-16

**Authors:** Christopher Bacon

**Affiliations:** 1 Associate Professor of Environmental Politics and Policy, Santa Clara University, Santa Clara, California, USA

I have reviewed the manuscript and the responses to my comments, and I am happy to change the status of my review to Approved.

Is the study design appropriate and does the work have academic merit?

Yes

Is the work clearly and accurately presented and does it cite the current literature?

Yes

If applicable, is the statistical analysis and its interpretation appropriate?

I cannot comment. A qualified statistician is required.

Are all the source data underlying the results available to ensure full reproducibility?

Yes

Are the conclusions drawn adequately supported by the results?

Partly

Are sufficient details of methods and analysis provided to allow replication by others?

Yes

Reviewer Expertise:

NA

I confirm that I have read this submission and believe that I have an appropriate level of expertise to confirm that it is of an acceptable scientific standard.

# Reviewer report: Reviewer response for version 1

**DOI:** 10.21956/openreseurope.17665.r42242

**Published:** 2024-09-14

**Authors:** Eric Fouilland

**Affiliations:** 1 Centre for Marine Biodiversity Exploitation and Conservation, Montpellier, Occitanie, France

The authors present findings based on data collected from 85 fish ponds in the Dombes region of France, sampled between 2007 and 2014. They concluded that implementing periodic one-year drying periods as an agroecological practice in fish farming enhances the mineralization of organic matter in sediments. This process initially results in high macrophyte coverage during the first year after drying but subsequently decreases, giving way to phytoplankton dominance. Fish yields were observed to peak in the second year following the drying period. Consequently, the authors suggest that a periodic one-year drying period, applied every two or three years, could optimize fish production while also promoting biodiversity conservation.

 The study is highly relevant as it addresses the balance between agricultural productivity and ecological sustainability, a major concern in contemporary agroecology. The conclusions drawn from this research provide valuable insights into how traditional fish farming practices can be optimized not only to maximize yield but also to support and enhance local biodiversity. By demonstrating that periodic drying can lead to improved fish production and healthier ecosystems, the study offers a practical approach that could be widely applicable to similar agroecological systems.

 However, some points need to be addressed:

 1- From the Methods section, it is indicated that five sampling periods were conducted, corresponding to the number of years since the last drying period of the ponds. This implies that all ponds were sampled after a one-year drying period. However, the authors also mention that "Y > 5 included all ponds that have been dry for five years or more," which appears to conflict with the earlier statement. This discrepancy needs to be clarified in the Methods section, as it could significantly impact the interpretation of the results and the study's overall conclusions.

 2-The methods used to measure organic matter, total nitrogen, and phosphate (in both water and sediment) require a more detailed description. Notably, no description was provided for the total nitrogen measurement in water, despite the data being used in Table 2. Further clarification is also needed regarding the « C :MO » conversion factor employed. Additionally, there is no mention of the quantity or species of fish introduced after the one-year drying period, nor is there any information provided about the initial water chemistry used to refill the ponds.

 3-The authors incorrectly refer to Figure 6 instead of Figure 5 when discussing fish yield and multivariate analysis in the Results section. Furthermore, contrary to the authors' statement in the Results section (under multivariate analysis), ponds that have been in water for two years are more centrally located in Figure 5 than ponds that have been in water for five years or more. I am also unconvinced by the linear regression presented in Figure 4 between Chl-a and cyanobacteria percentages in phytoplankton, as the R² value is lower than 0.5, indicating that less than 50% of the variability in cyanobacteria percentages is explained by increases in Chl-a. I would recommend removing Figure 4, as it does not contribute robust conclusions to the study. For Figure 5, it would be beneficial to include the percentage of variation in the original data for each axis and to replace the term "each pond class" with "the sampling years after the one-year drying period."

 4- It would have been valuable to describe the short-term dynamics of nitrate, nitrite, and total nitrogen in the water from the time of pond refilling. This data could have provided insights into the relative contributions of nitrogen from the water source versus nitrogen mineralized from sediment, thereby strengthening the authors' conclusion that "drying up favours incomplete mineralization of organic matter in the sediment."

 5- Additional information on fish consumption (macrophytes, phytoplankton, zooplankton) across different life stages and species would also be useful. This could help create a clearer understanding of the food web dynamics in the ponds over time, providing further explanations for the observed fish yields and the distribution of primary producers.

Is the study design appropriate and does the work have academic merit?

Yes

Is the work clearly and accurately presented and does it cite the current literature?

Yes

If applicable, is the statistical analysis and its interpretation appropriate?

Partly

Are all the source data underlying the results available to ensure full reproducibility?

Partly

Are the conclusions drawn adequately supported by the results?

Partly

Are sufficient details of methods and analysis provided to allow replication by others?

Partly

Reviewer Expertise:

microbial ecology

I confirm that I have read this submission and believe that I have an appropriate level of expertise to confirm that it is of an acceptable scientific standard, however I have significant reservations, as outlined above.

# Response

**Published:** 2024-09-24

**Authors:** Léo GIRARD

**Affiliations:** Isara, France

Dear reviewer, Thank you for your comments and suggestions. Here are our responses to the various points.

 1- From the Methods section, it is indicated that five sampling periods were conducted, corresponding to the number of years since the last drying period of the ponds. This implies that all ponds were sampled after a one-year drying period. However, the authors also mention that "Y > 5 included all ponds that have been dry for five years or more," which appears to conflict with the earlier statement. This discrepancy needs to be clarified in the Methods section, as it could significantly impact the interpretation of the results and the study's overall conclusions.

 Response: There is indeed a problem with the wording of this sentence. The Y>5 group includes fish ponds that have been dry out 5 years ago or more, not ponds that have been dry for 5 years. This has been corrected. As mentioned in the Materials and Methods section, fish ponds are drained every year to harvest fish and refilled within a few days/weeks. On average, every 4 to 5 years, they are drained for an entire production season, the dry out studied in this paper. The dataset include 85 fish ponds that were monitored several times between 2007 and 2014 (134 data in all). Each of these data is divided into a group according to the time since the last dry out.

 2-The methods used to measure organic matter, total nitrogen, and phosphate (in both water and sediment) require a more detailed description. Notably, no description was provided for the total nitrogen measurement in water, despite the data being used in Table 2. Further clarification is also needed regarding the « C :MO » conversion factor employed. Additionally, there is no mention of the quantity or species of fish introduced after the one-year drying period, nor is there any information provided about the initial water chemistry used to refill the ponds.

 Response: We have added information about total nitrogen concentration in water. Regarding the « C :MO » conversion factor, it’s based on the NF ISO 10694 standard (Determination of organic carbon and total carbon after dry combustion). This is the organic carbon to OM conversion index proposed in the standard we use in France for organic C determination. The fish species most commonly raised in extensive pond culture are described in the Materials and Methods section, along with average yields. Precise stocking rates vary between managers. The quality of the water used to fill the ponds is unknown. In most cases, they are filled by rainfall or water from an upstream pond.

 3-The authors incorrectly refer to Figure 6 instead of Figure 5 when discussing fish yield and multivariate analysis in the Results section. Furthermore, contrary to the authors' statement in the Results section (under multivariate analysis), ponds that have been in water for two years are more centrally located in Figure 5 than ponds that have been in water for five years or more. I am also unconvinced by the linear regression presented in Figure 4 between Chl-a and cyanobacteria percentages in phytoplankton, as the R² value is lower than 0.5, indicating that less than 50% of the variability in cyanobacteria percentages is explained by increases in Chl-a. I would recommend removing Figure 4, as it does not contribute robust conclusions to the study. For Figure 5, it would be beneficial to include the percentage of variation in the original data for each axis and to replace the term "each pond class" with "the sampling years after the one-year drying period."

 Response: We have corrected the reference to figure numbers. Ponds in Y2 are indeed centrally located as ponds from Y>5, but for different reasons. As we have seen from other parts of the results, year 2 is characterized by the presence of both primary producers, whereas ponds maintained in water for 5 years or more have less primary producers. But these two different situations are reflected in the same way on the first two dimensions of the multivariate analysis. We agree with the fact that less than 50% of the variability in cyanobacteria percentages is explained by increases in Chl-a. But we consider this to be an important element. High phytoplankton growth is generally associated with the dominance of cyanobacteria and all the negative effects this can have on ecosystem functioning.

 4- It would have been valuable to describe the short-term dynamics of nitrate, nitrite, and total nitrogen in the water from the time of pond refilling. This data could have provided insights into the relative contributions of nitrogen from the water source versus nitrogen mineralized from sediment, thereby strengthening the authors' conclusion that "drying up favours incomplete mineralization of organic matter in the sediment."

 Response: We agree that this could be interesting. However, we don't have the data to analyse the various sources and dynamics of inorganic nitrogen or phosphorus in the pond.

 5- Additional information on fish consumption (macrophytes, phytoplankton, zooplankton) across different life stages and species would also be useful. This could help create a clearer understanding of the food web dynamics in the ponds over time, providing further explanations for the observed fish yields and the distribution of primary producers.

 Response: Most of the species produced in ponds are omnivorous (carp, tench), detritivorous (roach) or piscivorous (pike and pikeperch). With rare exceptions, no herbivorous species are raised. Impacts on the trophic chain and the dynamics of primary producers are therefore mainly top-down. Benthic fish have a negative effect on macrophytes, both through turbidity linked to sediment resuspension and through mechanical destruction during foraging. The consumption of zooplankton also encourages the development of phytoplankton. Fish assemblages are fairly similar from one pond to the next, but also across the different years of the production cycle. This variable therefore has a minor effect on the dynamics observed in this paper in relation to the dry season.

# Reviewer report: Reviewer response for version 1

**DOI:** 10.21956/openreseurope.17665.r34752

**Published:** 2023-11-13

**Authors:** Christopher Bacon

**Affiliations:** 1 Associate Professor of Environmental Politics and Policy, Santa Clara University, Santa Clara, California, USA

The article entitled, “Drying out fish ponds, for an entire growth season, as an agroecological practice: maintaining primary producers for fish production and biodiversity conservation” contributes to an often overlooked area of agroecology by incorporating on-farm fish ponds. The consideration of the way that the pods relate to nutrient cycling and on-farm aquatic biodiversity offers an important and potentially innovative contribution to the literature and the scientific measurements and sample sizes are generally robust.

 Overall, I find the writing clear, the references well done and I think this will be of interest to readers of this journal. I do have several suggestions that could be useful for revisions, which I have organized according to the subsection below.

 Introduction 

1. A map / photo of some of these pondscapes could be a useful visual for readers.
2. Third paragraph first sentence - more biodiversity than which other environments? 
3. This same paragraph has a sequence of different propositions that either need more explanation and should be discussed in several separate paragraphs. There are ideas about why the pods were made commingled with ideas about how much biodiversity they sustain and if managed vs. unmanaged ones conserve more biodiversity.
4. First 2 paragraphs were strong, but I was a bit lost with all of these ideas in the third paragraph.
5. More clarity on why the dry out phase has been used used would be helpful.
6. Strong clear statement of study goals and hypothesis - I would preview these earlier in the introduction.

Methods 

1. Clear writing, robust research plan, and this section also helped further explain several questions I had in the introduction (see comments above). 

Results 

1. Generally clear, robust and well organized. 
2. A bit more detail in the figure captions explaining each figure would be helpful. 
3. Also reminders of the sample size and years covered. 

Discussion and conclusion 

1. Missing a summary of the results vs. in initial hypothesis.
2. Missing a border discussion about how this relates to the brief literature review engaged in the introduction and the agroecological management biodiversity over time and across land and water.. And, tie back into the discussion of agroecological principles.  
3. Conclusion is solid but could talk a bit more about generalizability to other places. 
4. Question - Why not broaden the focus to include agricultural ponds for irrigation not just for fish? 
5. As another broader implication that authors may want to consider in the discussion, or suggest for future research in the conclusion, is the extent to which this study could contribute to a broader research agenda related to the agroecology-based management of water systems.

Is the study design appropriate and does the work have academic merit?

Yes

Is the work clearly and accurately presented and does it cite the current literature?

Yes

If applicable, is the statistical analysis and its interpretation appropriate?

I cannot comment. A qualified statistician is required.

Are all the source data underlying the results available to ensure full reproducibility?

Yes

Are the conclusions drawn adequately supported by the results?

Partly

Are sufficient details of methods and analysis provided to allow replication by others?

Yes

Reviewer Expertise:

Agroecology

I confirm that I have read this submission and believe that I have an appropriate level of expertise to confirm that it is of an acceptable scientific standard, however I have significant reservations, as outlined above.

# Response

**Published:** 2024-09-24

**Authors:** Léo GIRARD

**Affiliations:** Isara, France

Dear reviewer, Thank you for your comments and suggestions. Here are our responses to the various points. **Introduction  **

1. A map / photo of some of these pondscapes could be a useful visual for readers.

We have added a map of fish ponds from the Dombes region, with two photos of a fish pond and a dry pond (Figure 1, Materials and Methods).

1. Third paragraph first sentence - more biodiversity than which other environments?

Than rivers, lakes or canals for example (other aquatic environments). These information have been added in the first sentence of the third paragraph.

1. This same paragraph has a sequence of different propositions that either need more explanation and should be discussed in several separate paragraphs. There are ideas about why the pods were made commingled with ideas about how much biodiversity they sustain and if managed vs. unmanaged ones conserve more biodiversity. 

We have reworked on this paragraph to clarify the different propositions.

1. First 2 paragraphs were strong, but I was a bit lost with all of these ideas in the third paragraph. 
2. More clarity on why the dry out phase has been used would be helpful. 

More sentences have been added in Materials and Methods (Study area and study sites) to clarify about dry out.

1. Strong clear statement of study goals and hypothesis - I would preview these earlier in the introduction.

The research question and hypotheses are derived from the introduction. It therefore makes more sense to place them at the end of the introduction, before the methodology section. 

**Methods  **

1. Clear writing, robust research plan, and this section also helped further explain several questions I had in the introduction (see comments above).

**Results  **

1. Generally clear, robust and well organized.
2. A bit more detail in the figure captions explaining each figure would be helpful.

Done.

1. Also reminders of the sample size and years covered.

We have added on sentence at the beginning of the result section to remind sample size and years covered. All these information are also available in Table 1. 

**Discussion and conclusion  **

1. Missing a summary of the results vs. in initial hypothesis.
2. Missing a border discussion about how this relates to the brief literature review engaged in the introduction and the agroecological management biodiversity over time and across land and water. And, tie back into the discussion of agroecological principles.

We have added some sentences at the beginning of the discussion both to come back to the elements of the introduction and the hypotheses, but also to summarize the results and introduce the discussion.

1. Conclusion is solid but could talk a bit more about generalizability to other places.
2. Question - Why not broaden the focus to include agricultural ponds for irrigation not just for fish?

It could indeed be interesting to extend the use of this practice to other types of ponds, including agricultural ponds for irrigation. Generally speaking, this could help to promote biodiversity and prevent future filling-in. We have added few words bout that at the end of the conclusion.

1. As another broader implication that authors may want to consider in the discussion, or suggest for future research in the conclusion, is the extent to which this study could contribute to a broader research agenda related to the agroecology-based management of water systems.

We have added a short paragraph at the end of the conclusion to broaden agroecological perspectives on the management of fish ponds and aquatic environments.
